# Low incidence of cannibalism among brood parasitic cuckoo catfish embryos

**DOI:** 10.1093/beheco/arad024

**Published:** 2023-04-08

**Authors:** Holger Zimmerman, Deryk Tolman, Martin Reichard

**Affiliations:** Institute of Vertebrate Biology, Czech Academy of Sciences, Květná 8, 603 00, Brno, Czech Republic; Institute of Vertebrate Biology, Czech Academy of Sciences, Květná 8, 603 00, Brno, Czech Republic; Helsinki Institute of Life Sciences, University of Helsinki, Yliopistonkatu 3, 00014 Helsinki, Finland; Institute of Vertebrate Biology, Czech Academy of Sciences, Květná 8, 603 00, Brno, Czech Republic; Department of Ecology and Vertebrate Zoology, University of Łódź, 12/16 Banacha street, 90-237 Łódź, Poland; Department of Botany and Zoology, Faculty of Science, Masaryk University, Kotlářská 2, 611 37, Brno, Czech Republic

**Keywords:** brood parasitism, cichlid fish, embryo predation, mouthbrooding, siblicide, sibling competition

## Abstract

Brood parasites have demanding needs of host resources. Brood parasitic offspring are highly competitive and frequently cause the failure of host broods and the survival of a single parasitic offspring. Accordingly, virulent brood parasites lay a single egg in the same host nest to avoid sibling competition. In the cuckoo catfish (*Synodontis multipunctatus*), which parasitize mouthbrooding cichlid fishes in Lake Tanganyika, the modes of host and parasite oviposition lead to frequent cases of multiple parasitism. We experimentally tested the prediction that multiple parasitism leads to frequent cannibalism among offspring. Cuckoo catfish embryos prey upon host offspring to obtain nourishment during their 3-week development in the host buccal cavity and may also consume conspecific embryos. The potential benefits of cannibalism in the system are, therefore, twofold; to decrease competition for limited resources (i.e., host brood with rich yolk sacs) and to directly obtain nourishment by consuming rivals. We found that cannibalism indeed provided measurable benefits in terms of increased growth of the cannibals, but cannibalism was rare and typically occurred once all host offspring had been consumed. This implies that cannibalism among cuckoo catfish embryos emerges to mitigate starvation rather than eliminate competition.

## Teaser

Offspring of brood parasites vigorously compete for host resources. Cuckoo catfish embryos feed on host offspring but only rarely resort to cannibalism, despite the ensuing benefits of decreased competition and direct energy provisions. Cannibalism among cuckoo catfish embryos emerges to mitigate starvation and not to eliminate competition.

## INTRODUCTION

Sibling interactions can have major implications for the fitness of individual offspring. Individuals often face a fitness trade-off between competing and cooperating with siblings (Roulin and Dreiss 2012). Obligate brood parasites, however, relegate parental care to their hosts, and parasitic offspring therefore find themselves among unrelated nestmates, eliminating the kin-selected benefits of cooperation in favor of competition. Highly virulent brood parasites, such as common cuckoos (*Cuculus canorus*) and honeyguides (*Indicator* spp.), limit host offspring survival through competitive exclusion (Soler 2017) or direct killing (Honza et al. 2007; Spottiswoode and Koorevaar 2012), in many cases resulting in the parasitic chick becoming the sole occupant of the nest. Concurrent (i.e., multiple or repeated) parasitism by two or more brood parasitic offspring within a single host clutch (Gloag, Fiorini, et al. 2012) is more commonly reported from brood parasites with a low impact on host offspring survival and condition (Martínez et al. 1998; Tuero et al. 2007; Goguen et al. 2011) than from virulent parasites (Marton 2021; Honza et al. 2022). This situation arises because high virulence is associated with increased competition for host resources, leading to poor survival prospects for the younger parasite (Moskát and Honza 2002; Spottiswoode 2013). Therefore, virulent brood parasites tend to lay only a single egg per host nest and avoid laying into already parasitized nests (Davies 2015).

The brood parasitic cuckoo catfish, *Synodontis multipunctatus*, from Lake Tanganyika evolved to exploit maternally mouthbrooding cichlid fishes (Sato 1986). Unlike other brood parasites, the cuckoo catfish and its hosts perform external fertilization, lay their eggs in an aquatic environment, and collect them from the substrate soon after oviposition for brooding. This mode of reproduction removes control over how many catfish eggs will parasitize host clutches, generating a high probability that host broods are parasitized by several eggs of one or more cuckoo catfish females despite the potential costs to individual parasite offspring. Parasitism involves intrusion by a group of cuckoo catfish into an ongoing cichlid host spawning, predation of some host cichlid eggs, and spawning of their own, smaller eggs among those of the host cichlid. When host females hastily collect their eggs to avoid predation, they may accidentally collect cuckoo catfish eggs. In addition to the reduction of the host clutch through adult catfish predation during spawning (Zimmermann et al. 2022), the parasites hatch earlier than and begin feeding on host offspring. Cuckoo catfish embryos possess specific morphological adaptations (in dentition, jaw morphology, and mouth position) to prey on host offspring (Cohen et al. 2019), and the entire host clutch may be consumed over the 2–4 weeks of development in the host buccal cavity (Sato 1986; Blažek et al. 2018).

The cuckoo catfish is a host generalist and, across host species, the average number of catfish eggs in host clutches is 3, ranging from 1 to 14 (Sato 1986; Takahashi and Koblmüller 2020). The nature of spawning by the cuckoo catfish and their hosts results in negligible control over the number of parasitic eggs collected by a host female, frequently leading to competition among related and non-related catfish embryos in the host buccal cavity. Some host species (e.g., *Tropheus moori*) only lay 10–20 eggs (Yanagisawa and Sato 1990) and multiple parasitism considerably constrains access to resources (i.e., host eggs and embryos) needed for successful development of multiple catfish embryos in the host buccal cavity. While elimination of intraspecific competitors, regardless of their relatedness, would improve access to the limited host clutch and improve individual catfish survival and growth, multiple parasitic offspring can successfully complete their development, and several well-developed juveniles have been reported to co-occur in a single host female (Sato 1986). Cuckoo catfish clutches are often of mixed parentage (H. Zimmermann, unpublished data) as host spawnings are intruded by groups of catfish (Blažek et al. 2022). Hence, as inclusive fitness benefits may favor the avoidance of sibling cannibalism (Penn and Frommen 2010), there is also scope for kin recognition and increased incidence of cannibalism in clutches with lower genetic relatedness (sensu Pfennig 1997, see Kilner 2005 and Rivers et al. 2012 for potential implications on brood parasitic reproduction).

Sibling cannibalism reduces competition for food and may directly act as a source of additional nutrition (Smith and Reay 1991; Pereira et al. 2017), as is observed in lamnid sharks (Miller et al. 2022). Given that high offspring densities facilitate sibling cannibalism (Polis 1981) and cuckoo catfish embryos must co-occur in the limited space of the host buccal cavity, we predicted cannibalism to be common among cuckoo catfish offspring. In this study, we experimentally simulated situations of conspecific competition across varying natural cuckoo catfish embryo densities and under constrained food (host embryo) availability.

## METHODS

### Fish housing and experimental setup

**Figure 1:**
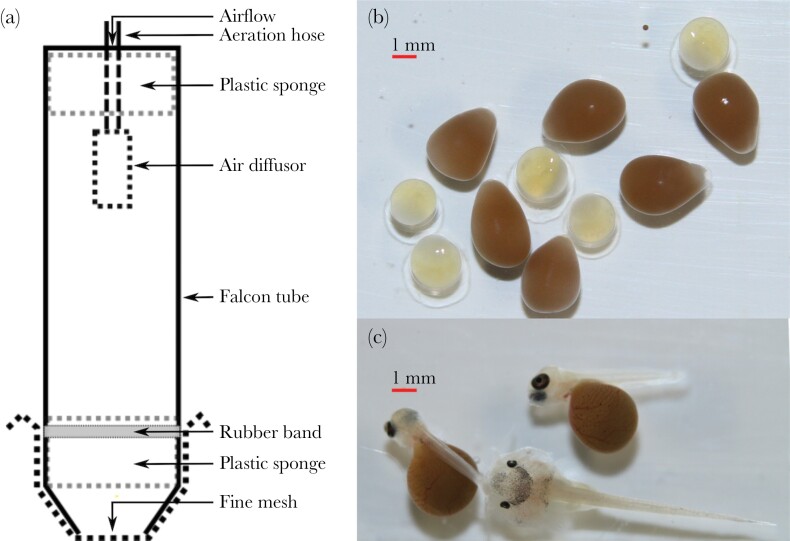
Schematic representation of the experimental setup (a). Pictures show a comparison of cuckoo catfish and host (*Gnathochromis pfefferi*) eggs on day 1 post-fertilization (b) and embryos of cuckoo catfish on day 6 post-fertilization with host offspring on day 7 post-fertilization (c).

The experiments were conducted between 31 January and 5 December 2021. Catfish and host cichlids (*Gnathochromis pfefferi*) used to obtain eggs for the experiment were housed in separate communal tanks (525 L), equipped with internal filtration and three or four shelters created from halved clay flower pots, at 25 °C (within the range of Lake Tanganyika natural water temperature) and a photoperiod of 13:11 h (light:dark). When a host female naturally spawned, we produced catfish eggs for the experiment using in-vitro fertilization (IVF) and placed them in custom-made tumblers. For the IVF, we gently stripped the eggs from one female into a large petri dish containing a small volume of water (approximately 10 mL) and immediately fertilized the eggs by stripping the sperm from three males while mixing the water with a Pasteur pipette. To produce a group of catfish embryos with mixed parentage, we repeated gamete collection in the same petri dish with more male and female catfish individuals. Between days 3 and 4 post-spawning, we collected the host eggs by gently removing them from host females using a jet of water from a Pasteur pipette. Until 6 days post-fertilization (6 dpf), host eggs and catfish eggs were incubated separately (see Figure 1b for a comparison of catfish and host eggs). Cuckoo catfish eggs hatched at 4 dpf and host eggs hatched at 5 dpf. The experiments started at 6 dpf by placing catfish and host embryos together in custom made tumblers resembling the narrow space of the host female buccal cavity. These tumblers consisted of a 50 mL Falcon tube (25 mm wide, 110 mm high, with a tapered end). The tapered end was cut off, leaving a 15 mm opening which was tightly covered using a fine mesh (0.1 mm). The egg movement inside the tumblers was restricted by a plastic sponge placed 10 mm above the mesh. Water flow through the tumblers was driven by an air stone located in the upper end of the tube and secured by a second layer of plastic sponge (Figure 1a). Proper aeration and imitation of host buccal cavity conditions was achieved by fine adjustment of the air flow to keep the embryos continuously gently bouncing to the mesh fabric in close contact to each other. Intraspecific embryo cohorts (i.e., conspecific embryos per replicate) were size-matched visually.

The experiment consisted of four treatments. Each treatment had a constant number of three host embryos and either zero, one, three, or six cuckoo catfish embryos. We chose three host embryos, a number which may represent an unusual food scarcity for catfish embryos early in development, for several reasons. First, although it characterizes a situation of low host spawning success, it is a feasible number for a host species with naturally low reproductive output (e.g., *Tropheus* spp.) when intruding adult cuckoo catfish prey on host eggs successfully. Second, the level of food scarcity used in this experiment is most likely natural also in host species with larger clutches (i.e., *G. pfefferi*) when they collect several parasite eggs. It is reported that cuckoo catfish embryos commonly eliminate entire host broods (Sato 1986; Blažek et al. 2018). This suggests that food scarcity will likely be an issue for parasite embryos (or juveniles) later in development. However, attempts of cannibalism at a late stage of development occasionally result in only a partial consumption of conspecific prey (personal observation) because of its large size and presence of sharp spines in the fins. These points raised ethical considerations and led us to study cannibalism early in the development. Two treatments were controls to estimate viability and survival of host embryos (zero-catfish treatment) and catfish embryos (one-catfish treatment) over the duration of the experimental trials. The three-catfish and six-catfish treatments resembled typical intermediate and high catfish densities. The numbers of host and parasite embryos were monitored every 24 h. Each replicate was terminated after 120 h to avoid catfish embryo mortality due to starvation. Host embryos obtained nourishment from their yolk sac over the entire experimental period. At the end of each replicate, the final number of surviving catfish embryos was recorded and every embryo was measured to the nearest 0.1 mm by photographing the embryos from above with a reference scale. Multiple measurements were taken for each embryo and only photographs where the embryo was positioned straight along the reference scale were used.

In total, 64 trials were conducted (4 treatments, 16 replicates each). In 16 trials (4 replicates of each treatment), the host embryos were one day older than the catfish embryos (7 vs. 6 dpf; see Figure 1c). In all other trials, host and catfish ages were matched (6 dpf). In eight replicates of each treatment, the catfish embryos were maternal half-sibs (eggs produced by a single female) and in the other eight replicates, catfish embryos were assigned haphazardly to their treatment tube from a large pool of IVF-produced embryos from multiple parents (typically 4–5 females and 10 males). We conducted experimental trials only when there were enough embryos available to set up all four treatments simultaneously (i.e., one experimental bout containing one replicate of each treatment). In the treatment with no catfish embryos, 85.42% ± 29.74% (mean ± SD) of host embryos survived until the end of the experimental period. In the treatment with one catfish embryo (i.e., no intraspecific competition/cannibalism), 87.5% ± 34.2% of catfish embryos survived. In one replicate where the catfish died (day 1 of the experiment), two cichlid embryos survived until the end (day 5 of the experiment), and one was consumed by the catfish before it died. This suggests catfish embryo developmental failure rather than a setup malfunction. In the second replicate with catfish mortality (between days 3 and 4 of the experiment), the single remaining host embryo also died.

### Statistics

All statistical analyses were conducted in the R environment, v. 4.1.2 (R Studio Team 2021). The assumptions and fit of linear models were inspected in the “DHARMa” package (Hartig 2021).

First, we tested whether treatment (i.e., relative prey density) affected the time needed for catfish to consume their foster siblings and the size of developing catfish embryos. To account for differences in within-treatment variances, we fitted two robust linear mixed effects models (function “rlmer” from the package “robustlmm,” Koller 2016). For one model, we used the time until all host embryos within the replicate were consumed as response variable and treatment (treatments with one, three, or six catfish embryos) as predictor variable. We additionally fitted the experimental bout as a random intercept to account for non-independence of data stemming from the same batch of embryos. For the second model, we used catfish embryo size (“catfish size”) as a response variable and treatment as the predictor variable. Additionally, we used “tumbler ID” as a random intercept to account for potential non-independence of data within each tumbler. Degrees of freedom were calculated using the non-robust model calculation (function “lmer,” package “lme4,” Bates et al. 2015). We used the three-catfish treatment as reference, but we also used the model with one-catfish treatment as the reference to investigate all pairwise contrasts among treatments.

To test whether catfish density affects the occurrence of cannibalism among catfish embryos, we analyzed catfish embryos during incubation (over 120 h). We performed a survival analysis using Cox proportional hazards (CoxPH) by fitting a CoxPH model (function “coxph” in the package “survival,” Therneau and Grambsch 2000). The response variable was catfish embryo survival, where every incidence of mortality split the sample in the input survival object according to a counting process approach (Sousa-Ferreira and Abreu 2019) to enable correct handling of a replicate with two cannibalistic events. Treatment (one-, three-, or six-catfish embryos), relatedness (half-sib or mixed parentage), and host embryo age (5 or 6 dpf) were included as independent variables.

To test the benefits of cannibalism, we compared the maximum size of catfish embryos between replicates with and without cannibalism. We fitted a generalized linear mixed model (GLMM, package glmmTMB, Brooks et al. 2017) with Gaussian error distribution and included the maximum catfish embryo size in each replicate as response variable. We included the occurrence of cannibalism (1, 0) and treatment (three or six catfish embryos) as well as their interaction as predictor variables. The age of host embryos (6 or 7 dpf) was modeled as a random intercept to account for a slightly larger embryo size in older hosts.

Finally, we tested whether the variation in body size within each experimental catfish embryo replicate affected the incidence of cannibalism. We fitted a generalized linear model (GLM) with binomial error distribution, where incidence of cannibalism (1, 0) was the response variable, and treatment (three or six catfish embryos) and the size difference between the largest and the smallest catfish embryo within a replicate were predictor variables. We included the age of host embryos (5 or 6 dpf) as a predictor variable as older (i.e., larger) host embryos might affect the food availability for the catfish embryos.

## RESULTS

### Incidence of sibling cannibalism

Six cannibalistic events were recorded out of 32 replicates in the treatments with multiple catfish embryos. This outcome included two replicates in the three-catfish treatment (prevalence of 12.50%) and three replicates (18.75%) in the six-catfish treatment, where either single (two replicates) or double (one replicate) cannibalism occurred. Cannibalism was validated by visual observation of an ongoing embryo consumption or the presence of catfish embryos in the gut. Only a single cannibalism act occurred when host embryos were still available as a food source (in the three-catfish treatment).

### Catfish embryos grew slower at higher density

**Figure 2:**
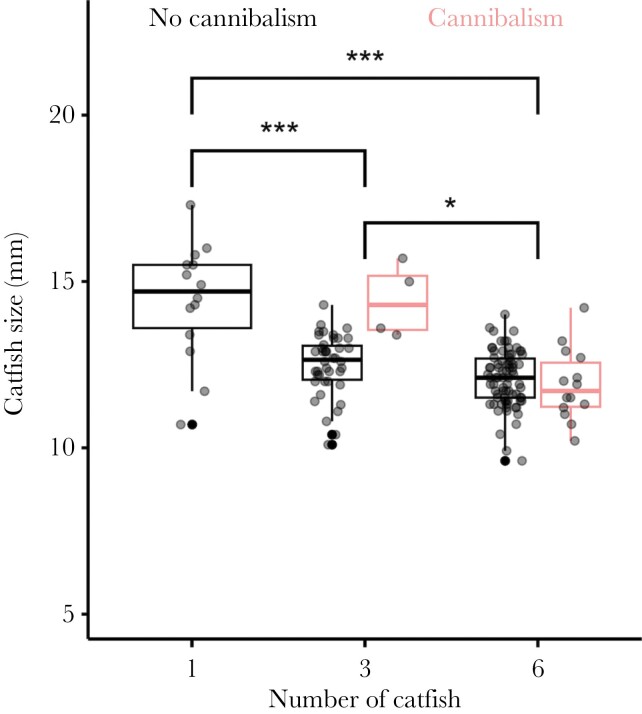
Body size of the cuckoo catfish embryos was larger when a single catfish embryo coexisted with three host embryos compared to treatments where three or six catfish competed. Gray dots show the measurements for individual catfish embryos within each replicate. Box plots show medians (horizontal lines), interquartile range (IQR, boxes) and the range of data within 1.5 IQR distances (whiskers). *P < 0.05, ***P < 0.001. Red lines represent replicates with the occurrence of cannibalism.

The growth of catfish embryos was negatively associated with their density (Figure 2) and corresponded well with the time they needed to consume all three host offspring. Host offspring survived until the end of the experiment in only two replicates (one 1-catfish replicate and one 3-catfish replicate) and time until all host embryos were consumed was significantly affected by catfish density (time until host embryos were consumed, mean ± SD: one-catfish-treatment = 78.9 ± 19.8 h, three-catfish-treatment = 43.5 ± 26.6 h, six-catfish-treatment = 28.5 ± 9.7 h; robust linear mixed model: one-catfish- vs. three-catfish-treatment, *t*_28.74_ = −8.69, *P* < 0.001, three-catfish- vs. six-catfish-treatment, *t*_28.27_ = −2.95, *P* < 0.01). Single catfish coexisting with their hosts without competitors (mean ± SD, 14.42 ± 1.77 mm) were significantly larger than catfish in the three-catfish treatment (12.68 ± 1.06 mm, robust linear mixed model: *t*_43.6_ = −5.78, *P* < 0.001) and six-catfish treatment (12.03 ± 0.87 mm, robust linear model: *t*_40.1_ = −8.20, *P* < 0.001). The catfish in the three-catfish treatment were significantly larger than catfish in the six-catfish treatment (robust linear mixed model: *t*_26.5_ = −2.71, *P* = 0.012).

### Catfish embryo survival was unaffected by their density

Survival of catfish embryos was high and did not differ between three-catfish and six-catfish treatments (mean ± SD, three-catfish: 95.8 ± 11.4%, six-catfish: 94.8 ± 10.0% survival; CoxPH: *n* = 55 trials, *z* = 0.800, *P* = 0.424). Neither relatedness within the catfish clutch (cannibalism occurred 3 times in the half-sib replicates and 3 times in the mixed-parentage replicates; CoxPH: *z* = −0.004, *P* = 0.996) nor the age of host embryos (CoxPH: *z* = 0.007, *P* = 0.994) affected catfish embryo survival.

### Cannibalism supported catfish embryo growth

**Figure 3:**
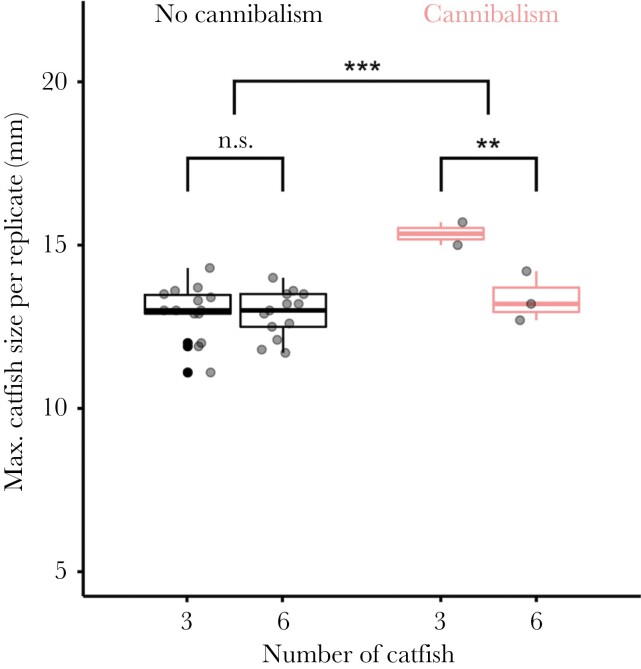
The largest catfish embryo in replicates with the occurrence of cannibalism was larger than in replicates without cannibalism. The faster growth rate due to cannibalism was more pronounced in the three-catfish treatment than in the six-catfish treatment. Gray dots represent measurements for each replicate. Box plots show medians (horizontal lines), interquartile range (IQR, boxes) and the range of data within IQR distances (whiskers). N.s. non-significant, **P < 0.01, ***P < 0.001.

Incidence of cannibalism was positively associated with the size of the largest catfish embryo in the replicate (mean size of the largest embryo in a clutch ± SD: with cannibalism = 14.16 ± 1.24 mm, without cannibalism = 12.93 ± 0.76 mm; GLMM: *n* = 32 trials, *z* = 4.39, *P* < 0.001; Figure 3). This outcome was independent of embryo density (three-catfish: 13.27 ± 1.13 mm, six-catfish: 12.98 ± 0.72 mm; GLMM: *n* = 32 trials, *z* = −0.29, *P* = 0.774), but there was a significant interaction between treatment and incidence of cannibalism which indicated that embryos in the six-catfish treatment benefited from cannibalism less than embryos in the three-catfish treatment (GLMM: *z* = −2.68, *P* = 0.007).

**Figure 4:**
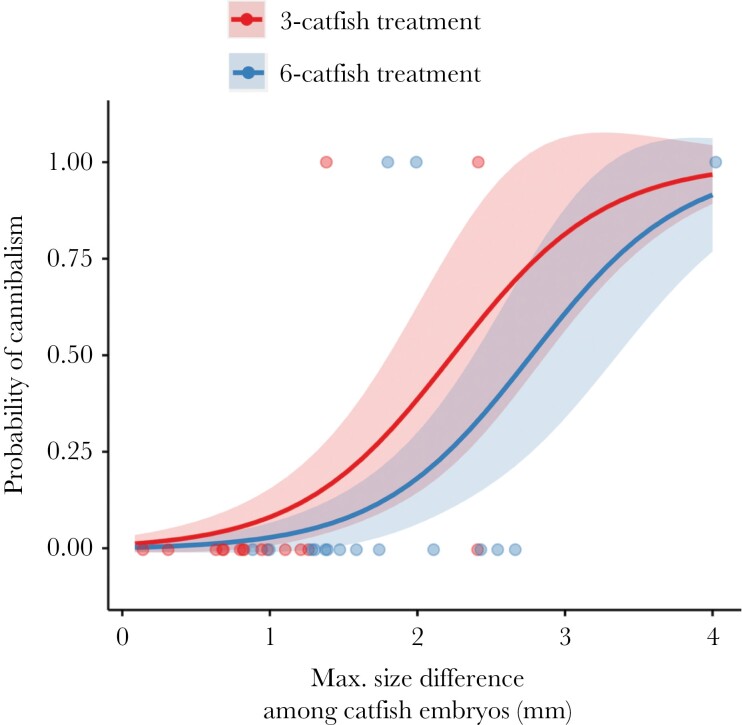
Cuckoo catfish embryos of replicates with cannibalism showed a larger within-replicate variation in body size than replicates without cannibalism. Lines represent the probability of cannibalism as predicted by a generalized linear model (GLM). Shaded areas represent the standard errors for estimates. Dots represent individual replicates.

Cannibalism was more common when the size difference between the smallest and the largest catfish embryo in the replicate was large (GLM: *n* = 32 trials, *z* = 2.169, *P* = 0.030; Figure 4). The mean (± SD) size difference was 2.28 ± 1.02 mm when cannibalism was recorded and 1.23 ± 0.66 mm when cannibalism did not occur. Neither the treatment (GLM: *z* = −0.782, *P* = 0.434) nor the age of host embryos (GLM: *z* = 0.237, *P* = 0.813) affected the incidence of cannibalism in our experiments.

## DISCUSSION

In this study, we demonstrate the existence of cannibalism among developing cuckoo catfish embryos. Despite measurable benefits in terms of increased growth of the cannibals, cannibalism was unexpectedly rare and typically occurred after all host offspring were consumed (five out of six cases). The mode of brood parasitism in the cuckoo catfish provides a catfish female with very limited control over how many of her offspring finally compete for limited prey density in the cichlid buccal cavity (Zimmermann et al. 2022). Although our experiments simulated food scarcity to facilitate the incidence of cannibalism among cuckoo catfish embryos, we designed experimental catfish embryo densities comparable to natural conditions (Sato 1986). Some host cichlids produce small clutches (< 20 eggs) while female cuckoo catfish regularly lay over 20 eggs to increase the success of parasitism. Multiple parasitism is therefore common and parasite offspring abundance varies considerably among parasitized broods (Sato 1986).

Offspring competition over resources is not uncommon and can lead to siblicide across a range of taxa (Morandini and Ferrer 2015). In highly virulent obligate avian brood parasites, chicks avoid competition for resources by eliminating their competitors (i.e., foster siblings) over parental provisions and monopolizing parental care (Kilner 2005). In fish, parental care is mostly restricted to guarding and sanitation (Smith and Wootton 1995; Balshine and Sloman 2011) and fish brood parasites primarily acquire offspring protection (Baba 1994). The cuckoo catfish is an exception, as their developing embryos use their foster siblings with extensive and nutrient-rich yolk sacs as a food resource (Cohen et al. 2019) and compete over access to prey from a shared host clutch. We confirmed the costs of competition among cuckoo catfish embryos through decreased growth rates in the treatments with more catfish embryos and hence greater competition over a standardized quantity of available prey. This situation is analogous to avian brood parasites, where ejecting or killing of foster siblings, or otherwise reducing host broods, has evolved to reduce or optimize competition for parental provisioning and increase parasite growth rates (Kilner et al. 2004; Gloag, Tuero, et al. 2012; Antonson et al. 2022).

Unlike in avian brood parasites, there is a second benefit to the elimination of conspecific competitors in the cuckoo catfish, because cannibalism provides an additional food resource. Although the statistical power of our analysis is restricted by the rare occurrence of cannibalism, our data suggest that preying on conspecifics indeed enhances the growth of cannibals. Nevertheless, observed cannibalism rates of 10–20% were lower than expected, appearing to represent a rather uncommon strategy of catfish embryos. This is a much lower rate compared to, for example, sand tiger shark embryos, which also benefit from embryo cannibalism in terms of accelerated growth. In contrast to the cuckoo catfish, the sand tiger shark embryos always perform intrauterine cannibalism first, before resorting to oophagy of unfertilised nutritional eggs (Gilmore et al. 2005).

One possible explanation for rare cannibalism among catfish embryos is their higher mobility compared to the embryos of host cichlids, which possess much larger yolk sacs and may represent much easier prey. Cohen et al. (2019) described regular interruptions of predatory attempts of the cuckoo catfish embryos caused by rapid movements of host cichlid embryos or the lack of suitable structures to grab onto. This explanation may account for why cannibalism was rare even at high catfish densities, which would otherwise favor sibling cannibalism in fish (Polis 1981). Finally, large within-clutch size differences further facilitate occurrence of siblicide and cannibalism (Smith and Reay 1991) and we found higher variance in catfish embryo size in replicates where cannibalism did occur. Whether the larger size difference indeed favored cannibalism or was its outcome could not be distinguished conclusively in our experimental design.

An alternative explanation for the scarcity of cannibalism in cuckoo catfish embryos comes from kinship discrimination. However, siblicide is predicted to evolve even among full siblings under certain conditions (Parker et al. 1989; Mock and Parker 1998) and competitive interactions must be intensified among siblings with a lower relatedness or among unrelated nestmates (Gloag and Beekman 2019). Fish are often capable of olfactorily distinguishing kin from unrelated individuals (Olsén 1992; Neff 2003; Penn and Frommen 2010) and kinship-related avoidance of cannibalism is known in fish (Liu et al. 2017). Less related broods of catfish in our experiment were not more inclined to cannibalism. Although our results suggest that cuckoo catfish embryos distinguish conspecifics from host embryos, catfish embryos may display size-related prey preference (i.e., prey on the smallest prey available first), regardless of their conspecific or heterospecific status. This would resemble patterns of kin discrimination in our experiment, when all host embryos were smaller than the smallest catfish embryo. Nevertheless, we think a preference for preying on heterospecifics is more likely because some common host species lay eggs up to three times larger than the eggs of our experimental host, *G. pfefferi* (Ahi et al. 2018; Cohen et al. 2019). Catfish embryos are usually smaller than embryos of these large-egg host species, yet their broods are commonly found to contain multiple coexisting catfish embryos (Sato 1986, our unpublished data). Further research is needed to explicitly test whether cuckoo catfish embryos can discriminate their kin.

While cannibalism conveyed greater growth rates and may enhance survival when host prey is scarce, we show that cannibalism and siblicide are not regular strategies of cuckoo catfish embryos. Instead of being employed to reduce competition over resources or gain direct fitness benefits through food acquisition, cuckoo catfish cannibalism appears to mitigate starvation.

## Data Availability

Analyses reported in this article can be reproduced using the data provided by Zimmermann et al. (2023).

## Notes

We thank Matej Polačik, Radim Blažek, Gernot Englmaier, Lukas Koch, Carl Smith and four anonymous referees for comments on the paper and Radim Blažek for his help in fish breeding and taking pictures of fish eggs and embryos.
